# Internet-Based Cognitive Behavioral Therapy for Chronic Fatigue Syndrome Integrated in Routine Clinical Care: Implementation Study

**DOI:** 10.2196/14037

**Published:** 2019-10-10

**Authors:** Margreet Worm-Smeitink, Arno van Dam, Saskia van Es, Rosalie van der Vaart, Andrea Evers, Michel Wensing, Hans Knoop

**Affiliations:** 1 Expert Center for Chronic Fatigue Department of Medical Psychology University Medical Centers, Vrije Universiteit Amsterdam Netherlands; 2 Department of Medical Psychology Radboud University Medical Center Nijmegen Netherlands; 3 Specialist Center for Complex Medically Unexplained Symptoms and Somatic Symptom Disorders Dimence Deventer Netherlands; 4 Tranzo School of Social and Behavioural Sciences Tilburg University Tilburg Netherlands; 5 GGZ-Westelijk Noord Brabant Institute for Mental Health Bergen op Zoom Netherlands; 6 PsyQ Somatiek en Psyche Parnassia Groep Amsterdam Netherlands; 7 Health, Medical and Neuropsychology Unit Institute of Psychology Leiden University Leiden Netherlands; 8 Department of Psychiatry Leiden University Medical Center Leiden Netherlands; 9 Department of General Practice and Health Services Research Heidelberg University Hospital Heidelberg Germany; 10 Radboud Institute of Health Sciences Radboud University Medical Center Nijmegen Netherlands; 11 Department of Medical Psychology Amsterdam University Medical Centers University of Amsterdam Amsterdam Netherlands

**Keywords:** eHealth, cognitive behavioral therapy, health plan implementation, chronic fatigue syndrome, attitudes

## Abstract

**Background:**

In a clinical trial, internet-based cognitive behavioral therapy (I-CBT) embedded in stepped care was established as noninferior to face-to-face cognitive behavioral therapy (CBT) for chronic fatigue syndrome (CFS). However, treatment effects observed in clinical trials may not necessarily be retained after implementation.

**Objective:**

This study aimed to investigate whether stepped care for CFS starting with I-CBT, followed by face-to-face CBT, if needed, was also effective in routine clinical care. Another objective was to explore the role of therapists’ attitudes toward electronic health (eHealth) and manualized treatment on treatment outcome.

**Methods:**

I-CBT was implemented in five mental health care centers (MHCs) with nine treatment sites throughout the Netherlands. All patients with CFS were offered I-CBT, followed by face-to-face CBT if still severely fatigued or disabled after I-CBT. Outcomes were the Checklist Individual Strength, physical and social functioning (Short-Form 36), and limitations in daily functioning according to the Work and Social Adjustment Scale. The change scores (pre to post stepped care) were compared with a benchmark: stepped care from a randomized controlled trial (RCT) testing this treatment format. We calculated correlations of therapists’ attitudes toward manualized treatment and eHealth with reduction of fatigue severity.

**Results:**

Overall, 100 CFS patients were referred to the centers. Of them, 79 started with I-CBT, 20 commenced directly with face-to-face CBT, and one did not start at all. After I-CBT, 48 patients met step-up criteria; of them, 11 stepped up to face-to-face CBT. Increase in physical functioning (score of 13.4), social functioning (20.4), and reduction of limitations (10.3) after stepped care delivered in routine clinical care fell within the benchmarks of the RCT (95% CIs: 12.8-17.6; 25.2-7.8; and 7.4-9.8, respectively). Reduction of fatigue severity in the MHCs was smaller (12.6) than in the RCT (95% CI 13.2-16.5). After I-CBT only, reduction of fatigue severity (13.2) fell within the benchmark of I-CBT alone (95% CI 11.1-14.2). Twenty therapists treated between one and 18 patients. Therapists were divided into two groups: one with the largest median reduction of fatigue and one with the smallest. Patients treated by the first group had a significantly larger reduction of fatigue severity (15.7 vs 9.0; *t*=2.42; *P*=.02). There were no (statistically significant) correlations between therapists’ attitudes and reduction in fatigue.

**Conclusions:**

This study is one of the first to evaluate stepped care with I-CBT as a first step in routine clinical care. Although fatigue severity and disabilities were reduced, reduction of fatigue severity appeared smaller than in the clinical trial. Further development of the treatment should aim at avoiding dropout and encouraging stepping up after I-CBT with limited results. Median reduction of fatigue severity varied largely between therapists. Further research will help understand the role of therapists’ attitudes in treatment outcome.

## Introduction

Chronic fatigue syndrome (CFS) is characterized by severe, persistent, and disabling fatigue. The fatigue is neither explained by the presence of a medical or psychiatric condition nor alleviated by rest. According to the revised Centers for Disease Control and Prevention (CDC) consensus criteria for CFS from 2003, 4 out of the following 8 additional symptoms should be present: problems with concentration and memory, sore throat, tender lymph nodes, headache, muscle pain, multi-joint pain, unrefreshing sleep, and postexertional malaise [1,2].

Cognitive behavioral therapy (CBT) for CFS is aimed at changing behavior and beliefs that maintain symptoms and can effectively reduce fatigue and disability [3,4]. Face-to-face CBT is intensive, requiring 12 to 16 sessions, and therapists need additional training to effectively deliver CBT for CFS [5,6]. Unfortunately, few therapists are trained for CBT for CFS, and therefore, treatment capacity is limited. To overcome this problem, Internet-based CBT (I-CBT) for CFS was developed, which was expected to demand less of therapist resources (ie, therapist time) and also be less burdensome for patients (no need to travel and working at own pace [7]). I-CBT for adult CFS patients was compared with a waiting-list condition in a randomized controlled trial (RCT) in a tertiary CFS treatment center in the Netherlands. It was found to lead to a significant reduction of fatigue and disability while taking approximately 45% less therapist time compared with that of face-to-face CBT (5:23/12:00 hours) [8]. As outcomes appeared less favorable than in face-to-face CBT and not all patients profited, I-CBT was subsequently embedded in stepped care: patients who were still severely fatigued or disabled after I-CBT could step up to face-to-face CBT. Stepped care was compared with care as usual, that was, only face-to-face CBT in a randomized controlled noninferiority trial. Stepped care was effective, and more efficient than care as usual, as it required less therapist time [9].

However, care that has proven to be effective within the context of an RCT, in a tertiary research center, is not necessarily equally effective in routine clinical care [10,11]. In case of CFS, it was found that face-to-face CBT could be provided in mental health care centers (MHCs) with the same magnitude of treatment effect as found in RCTs conducted in tertiary CFS research centers, although not all MHCs reached the benchmark [5,6]. It is not yet known if I-CBT for CFS implemented in routine care is effective. For I-CBT in other disorders, such as depression and anxiety, tinnitus, and irritable bowel syndrome, the first results suggest that it can be successfully implemented in routine clinical care, but more studies are needed [12]. Despite the evidence of the efficacy of electronic health (eHealth), it is incorporated in routine clinical care on a far smaller scale than expected [13,14]. Therefore, it is not yet known how I-CBT can best be embedded in routine clinical care. The first aim of this study is to investigate whether stepped care, comprising I-CBT followed by face-to-face CBT, can be delivered in routine clinical care as effective as in the RCT [9], with respect to treatment outcome.

Second, we are interested in the role of therapist variations on treatment outcomes in routine clinical care. The extent to which differences between the effectiveness of individual therapists explain variance in treatment outcome differs largely over studies. Less influence of therapist variation was associated with therapists being more experienced and the use of treatment manuals [15]. With respect to the treatment of CFS, in a large study in a specialist center, variance in outcome could not be explained by therapist factors. A possible explanation for this finding was that in specialized centers, therapists received the same training and supervision and had the same therapeutic orientation [16]. In a study evaluating the role of the therapist in routine clinical care for CFS (ie, face-to-face CBT), 21% of the variance in treatment outcome was explained by the therapist effect. Attitudes of individual therapists (ie, the attitude toward the use of treatment manuals) were associated with treatment outcome [17].

If therapists’ attitudes influence outcome, this provides an opportunity to enhance the efficacy of the treatment, as attitudes can be altered, for example, by experience or training [18,19]. We aimed to investigate if therapists’ attitudes toward treatment manuals also influenced outcome in implementation of I-CBT and stepped care for CFS in clinical routine care. Furthermore, we were interested in the role of attitudes of therapists toward eHealth. It was recently found that a positive attitude of the therapists toward eHealth was associated with sharing of more assignments with the therapist by the patient [20]. It was expected that attitudes of therapists influenced treatment outcome in our implementation study.

To answer our research questions, stepped care was implemented in 5 MHCs, with outpatient treatment centers in 9 different cities spread over the Netherlands. All CFS patients who were referred for CBT were offered I-CBT. If still severely fatigued or disabled after I-CBT, they were offered additional face-to-face CBT. We compared the reduction of fatigue and disability with the benchmark, which was, the effect of stepped care with I-CBT found in an RCT in a tertiary treatment center [9]. Both the effects of the I-CBT (the first step) and the full stepped care model were evaluated. We also explored outcome variations between individual therapists. More specifically, we determined if variation in treatment outcome of stepped care could be explained by therapists’ attitudes toward manualized treatment of CFS and the use of eHealth.

## Methods

### Design

This was an observational study with a pre- and posttreatment study design. Reduction of fatigue severity and level of disabilities were compared with a statistical benchmark, derived from a randomized controlled noninferiority trial for stepped care with I-CBT in a tertiary treatment facility [9] (registered in the Netherlands Trial Register as NTR4809). The latter study included 2 stepped care conditions, of which, the format of therapist feedback during I-CBT differed. In 1 condition, therapists' feedback was given at predefined time points. In the other condition, therapists' feedback was on demand. Both conditions were combined to represent the benchmark in this study (n=242).

MHCs that already offered face-to-face CBT for CFS for at least 1 year were asked to participate in the study. In the participating MHCs, I-CBT was implemented as a first step of stepped care, with additional face-to-face CBT as the second step.

The medical ethical committee of the Radboud university medical center ruled that the study did not fall under the scope of the Medical Research Involving Human Subjects Act (see Multimedia Appendix 1).

### Participants

#### Participating Mental Health Care Centers

Table 1 shows the characteristics of the participating MHCs.

The participating therapists were previously trained to deliver face-to-face CBT, during a 4-day training program in CBT for CFS followed by 2-week supervision for 1 year. All had gained experience in the face-to-face treatment of CFS [5].

In the context of the implementation of I-CBT, therapists had 2 additional training days. The first day focused on delivering I-CBT. The second day was scheduled after the first patients received treatment. The second training day focused on overcoming challenges that were met during delivering I-CBT and the process of stepping up to face-to-face CBT when necessary. Specific for face-to-face CBT after I-CBT is that although it follows a treatment manual [21], the starting point differs for each patient. To tailor the CBT to the patients’ process, therapists were trained to (1) identify what was already achieved during I-CBT and what was needed to further improve and (2) motivate patients to actually step up to face-to-face CBT. Therapists were trained to recognize and modify reduced expectations of the patient toward face-to-face CBT, after limited results during I-CBT.

**Table 1:** Characteristics of the participating mental health care centers.

Mental health care center	Treatment sites, n	Location site(s) in the Netherlands	Therapists
PsyQ Parnassia Groep	4	Central and west (the 4 largest cities of the Netherlands)	2-3 psychologists per site
PsyQ Lentis	2	Northeast	2 psychologists per site
PsyQ MET ggz	1	South	2 psychologists
GGNet	1	East	3 psychologists
GGz Westelijk Noord-Brabant	1	Central southwest	I-CBT^a^: 4 psychiatric nurses; face-to-face CBT^b^: 3 psychologists

^a^I-CBT: internet-based cognitive behavioral therapy.

^b^CBT: cognitive behavioral therapy.

#### Participating Patients

All adult patients referred for the treatment of CFS could participate if the following criteria were met: (1) a physician had concluded that the patient suffered from severe and disabling fatigue not explained by a known somatic or psychiatric condition; (2) the 2003 CDC consensus criteria for CFS were met (ie, severe, disabling fatigue was present, lasting for at least 6 months, accompanied by at least 4 out of 8 additional symptoms) or patients met criteria for *idiopathic chronic fatigue* (ICF) *syndrome* (ie, reported severe and persistent fatigue but did not meet all CDC criteria, <4 additional symptoms, or less impact on daily functioning [22]). Inclusion criteria for both patient groups were the presence of severe and persistent fatigue, as indicated by a score of ≥35 on the Checklist Individual Strength (CIS) Fatigue Severity Subscale [23], limitations in functioning according to the Short Form-36 (SF-36) Physical or Social Scale <65 [24], or both >65 but the patient was limited in daily functioning according to a clinical interview, for example, worked less; and (3) the patient had computer and internet access. There were no specific exclusion criteria.

### Intervention

All patients were offered I-CBT as a first step of treatment. If patients met step-up criteria after 6 months of I-CBT (still severely fatigued as indicated by CIS fatigue severity ≥35 or limited in functioning as indicated by SF-36 Physical or Social Functioning Scale ≤65), face-to-face CBT was offered.

Both forms of CBT were based on a treatment manual [21] that has been used in RCTs testing the efficacy of I-CBT and face-to-face CBT for CFS [25,26]. CBT comprises interventions aimed at changing behavior and cognitions that maintain CFS symptoms. It starts with setting concrete goals in terms of activity, which when reached, imply recovery (ie, no longer severely fatigued and disabled). Patients learn to establish a fixed sleep-wake cycle to recognize and modify dysfunctional cognitions and redirect their focus on symptoms to other matters. After this, patients start with a graded (physical) activity program, usually walking or cycling. The activity program is tailored to the patient, based on their activity pattern. The increase in activity is time contingent, irrespective of symptoms. In the same manner, social and mental (eg, reading) activities are increased and personal goals are attained.

The efficacy of I-CBT was tested in an RCT [8], and it is described in detail elsewhere [27]. The intervention comprises 7 modules corresponding to the different elements of the face-to-face protocol. After the first module is finished (*getting started and goal setting*), the following 5 modules become accessible. These modules were as follows: *regulate sleep-wake cycle*, *helpful beliefs about fatigue*, *how to communicate with others about CFS*, and *gradually increasing my activity*. When the sixth module *reaching my goals step by step* is finished, the seventh module opens (*evaluation and the future*). The duration of the modules differed, and the patient could work through them at his or her own pace for 6 months. Therapists were instructed to provide feedback weekly in the first month and at least fortnightly in the following 5 months. Patients were sent reminders if they did not report on their progress according to the aforementioned schedule. Therapists could respond to an assignment the patient completed or send an email via the platform. Feedback was aimed at helping the patients change their behavior and cognitions according to the principles they learned in the modules [27].

Accessibility differed between MHCs. A total of 3 MHCs used the same platform as the tertiary treatment center, and 2 MHCs incorporated the content of the intervention into their own eHealth portal. All used the same treatment content.

After 6 months of I-CBT, patients were invited for an face-to-face evaluation session and offered additional face-to-face CBT when they still met the aforementioned step-up criteria. The face-to-face CBT was delivered according to the treatment manual, although the starting point was tailored to the needs of the patient, as some cognitions and behaviors already changed during I-CBT. During the evaluation session, it was examined which cognitions and behavior remained dysfunctional and would be the focus of the additional face-to-face CBT. The number of CBT sessions could vary, and the expected maximum duration was 6 months. The face-to-face CBT therapist was preferably the same therapist who delivered I-CBT. In 1 MHC, this was not possible, as I-CBT was provided by a psychiatric nurse, whereas the face-to-face CBT was provided by a psychologist. Information on the course of the treatment was given to the psychologist.

### Measures

Before the start of the study, all therapists were asked to complete questionnaires assessing their attitudes toward the manualized treatment and the use of eHealth.

Patient data were collected by therapists. Some MHCs used digital questionnaires, some used pencil and paper versions of the outcome measures. The therapists gave each participant a unique research number and entered the relevant data into a spreadsheet, listed by research number and without personal information, except for age and sex. The spreadsheet was accessible to the researcher.

For patients who received the full stepped care, outcomes were measured at baseline, after I-CBT (6 months after start), and directly after face-to-face CBT (posttreatment assessment). For patients who received I-CBT only, the outcome directly after I-CBT was used as posttreatment assessment.

#### Fatigue Severity

Fatigue severity was measured with a fatigue questionnaire, the 20-item CIS [28], that measured different aspects of fatigue. The Fatigue Severity subscale is used to assess the level of fatigue and comprises 8 items, scored on a 7-point Likert scale (range 8-56, higher scores indicate more severe fatigue). The CIS has proven to be reliable (Cronbach alpha for fatigue severity subscale ranges between .69 and .94 [23,28]) and valid and has been used extensively in CFS research as outcome measure [28].

#### Functioning

Physical and social functioning was measured with the Medical Outcomes Survey SF-36 [24], a reliable and valid instrument to measure health status [29]. Cronbach alpha of the Dutch version is .92 for the Physical Functioning Subscale and .71 for the Social Functioning Subscale [30]. The Physical Functioning Subscale assesses physical functioning with 10 items. Scores on the scale range from 0 to 100, with higher scores indicating better physical functioning. The Social Functioning Subscale assesses impairment in social functioning with 2 questions scored. Total scores range from 0 to 100; higher scores indicate better social functioning.

Impairment in daily functioning was measured with the Work and Social Adjustment Scale (WSAS [31]). This scale assesses functioning at work, in home management, and in social and leisure activities, using 5 items, on a scale ranging from 0 to 8 (range of total score is 0-40, with higher scores indicating more impairment). The Dutch version of the WSAS is validated in CFS patients; Cronbach alpha is .89 [32].

#### Additional Symptoms

The number of additional CDC symptoms [1] was registered during the interview. Some therapists used a pen-and-paper checklist that systematically checked existing symptoms and a minimal duration of 6 months. The symptom maximum was 9, as concentration and memory problems were recorded separately.

#### Therapists’ Attitudes

Therapists’ attitudes toward the use of treatment manuals were assessed with a questionnaire developed by Addis et al [33] to measure attitudes of psychologists. It measures 2 constructs: (1) *Positive Outcome* (7 items), which reflects the attitude that manuals can contribute positively to treatment outcome (Cronbach alpha=.93) and (2) *Negative Process* (10 items), which reflects the attitude that the use of treatment manuals negatively influence the treatment process (Cronbach alpha=.80). A Dutch version was used, with a 6-point scale scored from 0 to 5 (positive outcome range 0-35, negative process range 0-50) [17].

Attitudes toward the use of eHealth were measured with an 18-item version of the eHealth attitude list [19]. The scale *P* *ossibilities of eHealth* contains 7 items (5-point Likert scale, range 7-35), and higher scores reflect the attitude that eHealth can be valuable. The scale *eHealth Negative Effects* contains 9 items (5-point Likert scale, range 9-45), and higher scores represent the attitude that eHealth poses a threat to the therapy process. The scale *Computer Competence* contains 2 items (5-point Likert scale, range 2-10), and a higher score indicates that the therapist feels competent in using computers. Structural and internal validity and internal consistency are good (Cronbach alpha between .83 and .89) [19,20].

### Statistical Analyses

Analyses were conducted after imputation of missing primary outcomes after I-CBT and missing primary and secondary outcomes at posttreatment (after face-to-face CBT or after I-CBT for patients who did not receive face-to-face CBT), using multiple imputations and assuming data were missing at random. A total of 20 imputations were performed. CIS, SF-36 Physical functioning and Social functioning, and total score on the WSAS at baseline and posttreatment were entered as predictors and variables to impute.

In the dataset used for the statistical benchmark [9], the CIS and the SF-36 Physical functioning were imputed in the same manner. For the purpose of this study, WSAS and SF-36 Social functioning scale were imputed likewise in the dataset.

Imputation and statistical analyses were performed with IBM SPSS version 22.

#### Treatment Effects

Treatment effects were tested with paired samples *t* tests for each outcome measure. To answer the primary research question, it was determined whether the change score between baseline and posttreatment assessment fell within the 95% CI of the change scores found in the RCT performed in the tertiary CFS research center (n=242) [9].

To specifically explore the efficacy of I-CBT implemented in the MHCs, fatigue severity before and after I-CBT was compared using a paired samples *t* test, and the change score in fatigue severity of I-CBT was compared with the benchmark of I-CBT from the RCT.

Uncontrolled effect sizes (within group Cohen *d*) were calculated for the CIS fatigue severity subscale, SF-36 physical and social functioning, and for the WSAS total score [34]. This was done by dividing the difference between the mean at baseline and postassessment by a pooled standard deviation (√[SD_pre_^2^+SD_post_^2^]/2). CIs were calculated following Hunter and Schmidt [35].

A sensitivity analysis was performed, in which missing data on CIS fatigue severity were not imputed but replaced by the maximum score (56). This was done with the assumption that patients who had no postassessment deteriorated. In the benchmark study, there were no missing CIS values.

#### Proportion of Patients With Clinically Significant Improvement in Fatigue Severity

A clinically significant improvement in fatigue severity was defined as a statistically Reliable Change Index (RCI) of >1.96 SD in CIS fatigue severity [36], in combination with a CIS fatigue severity score of <35 on postassessment. The score of <35 indicates that the patient is no longer severely fatigued [28]. The reliability of the CIS used in the RCI calculation was 0.88 [9,23]. An RCI of >1.96 SD means it can be assumed with a confidence of 95% that the improvement in CIS fatigue severity is not caused by unreliability of the measure but represents a true change.

#### Subgroup Analyses

To facilitate comparison with the benchmark study, in which patients with ICF syndrome were not included, change scores were calculated for the subgroup of patients in implemented stepped care who did meet the CDC criteria for CFS.

#### Therapists' Attitudes and Treatment Outcome

A mixed-models approach was planned to investigate to what extent variance in treatment effect could be explained by the therapists and their attitudes toward I-CBT and the use of treatment manuals. If the number of patients per therapist would be too low, variations in treatment outcome between therapists would be explored by dividing the therapists in 2 groups using a median split based on CIS fatigue change scores. Reduction in fatigue severity in patients of both groups would be compared using a *t* test.

To explore if therapists’ attitudes toward I-CBT and treatment manuals were related to treatment outcome, correlations were calculated between the therapists’ attitude subscale scores and the mean change score in fatigue severity of their patients. When no more than 1 item on an attitude subscale was missing, the missing value was replaced with the mean score on that subscale.

## Results

### Overview

Data of all 100 participants were analyzed. Postassessment data of the primary outcome measure (CIS fatigue severity) of 87 participants (87/100, 87.0%) was present.

**Figure 1:**
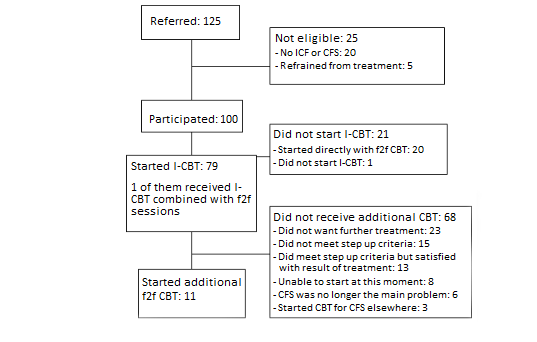
Flowchart. CBT: cognitive behavioral therapy; CFS: chronic fatigue syndrome; f2f: face-to-face; I-CBT: internet-based cognitive behavioral therapy; ICF: idiopathic chronic fatigue.

From October 2014 to December 2016, 125 patients were referred for treatment for CFS (Figure 1). Of them, 100 were eligible to enter the study and were included, 20 had no CFS (were not severely fatigued or had another diagnosis that explained the presence of fatigue), and 5 did not want treatment.

Out of the 100 eligible patients, 73 met all CDC criteria for CFS, 10 patients had <4 additional symptoms, for 14 patients the number of additional symptoms was unknown, and 3 patients were not severely impaired in functioning according to the SF-36 (both social and physical functioning ≥65) but reported severe impairment during the clinical interview.

In total, 79 patients started with I-CBT as intended, whereas 20 patients started directly with face-to-face CBT (20%). For 7 patients, it was reported that the patient preferred face-to-face CBT; for the others, reasons were not reported. In the benchmark study, 14 patients (14/242, 5.8%) who intended to start with I-CBT, started directly with face-to-face CBT [9], whereas 119 patients (119/766, 15.5%) eligible to enter the trial refused because they preferred face-to-face CBT. In the implementation study, 1 patient did not start treatment. For 24 patients (24/79, 30%), the therapist assumed dropout during I-CBT (no response and no new log-ins observed, or the patient explicitly reported to have stopped).

After I-CBT, 15 patients (15/79, 19%) did not meet the step-up criteria (were no longer severely fatigued or disabled). For 16 patients (16/79, 20%), it is not known whether step-up criteria were met, as post–I-CBT assessment scores were missing. The other 48 patients (48/79, 61%) with posttreatment data met step-up criteria. In total, 11 patients stepped up to face-to-face CBT (11/48, 23%). Reasons for not stepping up are given in the flowchart (Figure 1). In the benchmark study, 172 patients (172/242, 71.1%) met step-up criteria after I-CBT. Of them, 85 (49.4%) stepped up to face-to-face CBT [9].

### Patient Characteristics

Baseline characteristics are shown in Table 2. The proportion of female patients was larger than that in the stepped care arms of the RCT [9], with which the data were compared, which had a relatively low proportion of females [8]. Age in years, fatigue severity, and physical functioning did not significantly differ between this study sample and the benchmark sample (see Table 2). Social functioning and impairment in daily functioning were significantly worse in the MHC sample, whereas patients in the benchmark sample reported significantly more additional CDC symptoms.

**Table 2:** Baseline characteristics of this study and the benchmark study.

Baseline characteristic	MHC^a^ sample—stepped care in routine clinical care	Benchmark sample—stepped care in RCT^b^ (n=242)	Difference between samples			
			Chi-square (*df*)	*t* test (*df*)	Mann-Whitney *U* test	*P* value
Proportion female, n (%)	78 (78)	147 (60.7)	9.4 (1)^c^	—^d^	—	.002
Age (years; n=100), mean (SD)	37.4 (11.9)	36.9 (12.5)	—	−0.345 (340)	—	.73
Fatigue severity (CIS^e^; n=100), mean (SD)	49.6 (5.2)	50.5 (4.9)	—	1.580 (340)	—	.12
Physical functioning (SF-36^f^; n=98), mean (SD)	60.3 (21.3)	61.4 (19.7)	—	0.455 (338)	—	.65
Social functioning (SF-36; n=96), mean (SD)	37.2 (23.8)	44.0 (23.7)	—	2.365 (336)	—	.02
Impairment daily functioning (WSAS^g^; n=92), mean (SD)	25.6 (6.5)	23.2 (6.7)	—	−2.957 (332)	—	.003
Number of additional CDC^h^ symptoms (0-9; n=86), median (IQR^i^)	6 (3)	7 (2)	—	—	7703	<.001

^a^MHC: mental health care center.

^b^RCT: randomized controlled trial.

^c^n=342.

^d^Not applicable.

^e^CIS: Checklist Individual Strength.

^f^SF-36: Short Form-36.

^g^WSAS: Work and Social Adjustment Scale.

^h^CDC: Centers for Disease Control and Prevention.

^i^IQR: interquartile range.

### Treatment Effect

As shown in Table 3, patients significantly improved on all outcomes. Compared with the benchmark, the decrease in fatigue severity (CIS) is lower in the MHC sample, as the change score falls outside the 95% CI of the change in the benchmark study. For physical functioning and social functioning, the change scores fall within the CI of the benchmark. For limitations measured with the WSAS, the change score is above the CI of the benchmark.

For the sensitivity analysis, the CIS fatigue severity postassessment scores of all 13 patients with missing data were replaced with the maximum score of 56. This reduced the fatigue change score to 10.1, which was still a significant improvement (*t*=7.2, *P*<.001), that falls outside this CI.

### Proportion of Patients With Clinically Significant Improvement in Fatigue Severity

Data were missing for 13 patients, and it was assumed that they did not show a significant change in fatigue severity. Of the 100 patients, 37 (37/100, 37%) had a reliable and clinically significant improvement in fatigue severity and were no longer severely fatigued. In the stepped care conditions in the tertiary center, no CIS data were missing and 110 (110/242, 45.5%) had a clinically significant improvement in fatigue. The difference between the 2 improvement rates was not significant; χ^2^_1_(N=342)=2.1 and *P*=.15.

### Subgroup Analyses

When restricting the analyses to patients who met CDC criteria for CFS (n=73), scores on all outcomes were significantly improved (Table 3). The change scores on all outcomes were larger than that in the total group. All scores fall within the CIs of the tertiary treatment center, except for the change score in limitations (WSAS), which falls outside the CI of the benchmark. A post hoc analysis of variance comparing the CFS group with the ICF group (n=14) and the group for which, because of missing data, it was unknown whether the diagnosis was CFS or ICF (n=13), showed a group effect. CFS patients had a significantly larger reduction in fatigue severity than the patients with diagnosis unknown. Differences between the ICF group and both other groups were not significant.

The proportion of patients with a reliable and clinically significant improvement in fatigue severity was 29 out of 73 (40%). Data of 11 patients were missing, and no improvement was assumed for them.

**Table 3:** Treatment effect.

Outcome measure and selected group		n (%)	Treatment		Change score (95% CI)	*t* test	*P* value	Effect size (*d*)
			Pre	Post				
**Fatigue severity (CIS^a^)**								
	MHC—total group^b^	100 (13)	49.6	37.0	12.6 (9.7 to 15.5)	8.5	<.001	1.14 (0.84-1.44)
	MHC—CFS only^c^	73 (15)	50.2	35.9	14.4 (11.0 to 17.8)	8.3	<.001	1.31 (0.95-1.67)
	Benchmark^d^	242 (0)	50.5	35.6	14.9 (13.2 to 16.5)	17.8	<.001	1.47 (1.27-1.67)
**Physical functioning (SF-36^e^)**								
	MHC—total group	100 (32)	60.1	73.5	−13.4 (−18.1 to −8.7)	−5.6	<.001	0.62 (0.34-0.91)
	MHC—CFS only	73 (33)	59.0	75.4	−16.4 (−21.9 to −10.9)	−5.9	<.001	0.76 (0.42-1.10)
	Benchmark	242 (5)	61.4	76.6	−15.2 (−17.6 to −12.8)	−12.3	<.001	0.71 (0.53-0.90)
**Social functioning** **(SF-36)**								
	MHC—total group	100 (34)	37.0	57.5	−20.4 (−27.9 to −12.9)	−5.4	<.001	0.73 (0.44-1.01)
	MHC—CFS only	73 (34)	33.7	59.3	−25.6 (−34.0 to −17.2)	−6.0	<.001	0.96 (0.61-1.30)
	Benchmark	242 (5)	44.0	65.5	−21.5 (−25.2 to −17.8)	−11.4	<.001	0.84 (0.65-1.02)
**Limitations (WSAS^f^)**								
	MHC—total group	100 (40)	26.1	15.8	10.3 (7.8 to 12.7)	8.3	<.001	1.08 (0.78-1.38)
	MHC—CFS only	73 (40)	26.5	15.0	11.4 (8.6 to 14.3)	7.9	<.001	1.24 (0.88-1.59)
	Benchmark	242 (14)	23.2	14.6	8.6 (7.4 to 9.8)	14.0	<.001	0.99 (0.81-1.18)

^a^CIS: Checklist Individual Strength.

^b^MHC (mental health care center)—total group: all 100 participants, regardless of meeting Centers for Disease Control and Prevention criteria for chronic fatigue syndrome.

^c^CFS (chronic fatigue syndrome) only: subgroup of 73 participants that met Centers for Disease Control and Prevention criteria for chronic fatigue syndrome.

^d^Benchmark: patients who were allocated to the stepped care arms of the randomized control trial, all meeting Centers for Disease Control and Prevention criteria for chronic fatigue syndrome.

^e^SF-36: Short Form-36.

^f^WSAS: Work and Social Adjustment Scale.

Of the 80 patients who intended to start I-CBT, 64 had completed the post–I-CBT assessment, and 16 CIS fatigue severity scores were imputed. The mean CIS fatigue score after I-CBT was 36.7, which was on average 13.2 points lower than that at the preassessment (95% CI 9.8-16.5; *t*=7.8; *P*<.001). This change score falls within the 95% CI of the benchmark from the patients who followed I-CBT in tertiary treatment center (95% CI 11.1-14.2). A sensitivity analysis was performed by replacing all missing CIS fatigue scores post–I-CBT with the maximum fatigue score (56). This resulted in a change score of 8.7 (95% CI 5.6-11.7; *t*=5.6; *P*<.001), which fell below the benchmark.

### Therapists’ Attitude and Treatment Outcome of Fatigue Severity

In total, 25 therapists participated in the study. Of them, 15 therapists treated at least one patient and completed attitude questionnaires, 5 treated at least one patient but did not complete these questionnaires, and 5 completed the questionnaires but did not treat a patient. The number of patients per therapist varied from 1 to 18.

The 20 therapists who treated patients were ranked based on the median fatigue severity change score of their patients. The 10 therapists with the lowest median change score treated 43 patients, with a mean change score in fatigue severity of 9.0 points. The 10 therapists with the highest median change scores treated 57 patients who had a mean change score of 15.7. The difference between these means was significant (see Table 4). No differences between the 2 groups were found in therapists’ attitudes, except for the *Computer Competence* scale. The therapists with higher median fatigue change scores had significantly higher scores on *Computer Competence*. Furthermore, differences on baseline characteristics (fatigue, physical functioning, social functioning, number of additional CDC symptoms, level of limitations, age, and sex) of the patients treated by both therapist groups were compared using *t* tests and chi-square tests, and no difference was significant (*P*=.07-.93).

**Table 4:** Therapists attitude and treatment outcome (data of all 20 therapists who completed the questionnaires provided and of whom, 5 had not treated a chronic fatigue syndrome patient during the study).

Variable		All therapists	Therapists		Statistical difference between therapists with high and low median	
			With high median	With low median	*t*	*P* value
Patients treated^a^, n (%)		100 (87)	57 (48)	43 (39)	—^b^	—
Change score in fatigue severity, mean		12.7	15.7	9.0	2.42	.02
**Attitude eHealth^c^, mean (SD)**						
	Possibilities of eHealth scale (range 9-45)	28.0 (2.6)	28.6 (2.5)	25.8 (2.5)	−2.05	.06
	eHealth Negative Effect Scale (range 7-35)	25.2 (5.9)	25.6 (6.3)	26.8 (4.6)	0.38	.71
	Computer Competence Scale (range 2-10)	8.1 (1.7)	8.7 (1.3)	7.0 (1.4)	−2.28	.04
**Attitude manualized treatment, mean (SD)**						
	Positive outcome (range 0-35)	24.9 (4.1)	24.8 (4.7)	24.8 (4.1)	0.00	>.99
	Negative process (range 0-50)	14.6 (7.4)	14.9 (8.1)	13.8 (7.4)	−0.25	.80

^a^Number of patients with complete data.

^b^Not applicable.

^c^eHealth: electronic health.

The correlation between the fatigue severity change score per therapist and the attitude subscales were as follows: .363 for *Possibilities of eHealth* (*P*=.18), −.092 for *eHealth Negative Effect* (*P*=.75), .271 for *Computer Competence* (*P*=.33), .119 for *Positive Outcome* of treatment manuals (*P*=.67), and −.186 with *Negative Process* of treatment manuals. None of the correlations were significant (*P*=.51).

## Discussion

### Principal Findings

This is one of few studies that implemented and evaluated eHealth in routine clinical care. It shows that I-CBT embedded in stepped care for CFS can lead to a significant reduction in fatigue severity and limitations in routine clinical care. The outcomes were compared with those of the same treatment format delivered in an RCT, in a tertiary treatment center for CFS, the benchmark. Outcomes for limitations in functioning were similar in both settings or even better in the implemented care. Nevertheless, the decrease in fatigue severity was smaller in the MHCs. Compared with the benchmark, relatively fewer patients had a clinically significant improvement in fatigue severity (reliable change in fatigue and no longer severely fatigued) after stepped care, but the difference was not statistically significant. It should be noted that the improvement rate in the benchmark is also lower than what was previously found in the tertiary treatment center in an face-to-face treatment [3]. Although these studies are not entirely comparable, this suggests that there is probably room for improvement in the delivery of this treatment by MHCs in routine clinical care. We will elaborate on this in the future directions section.

We found that I-CBT, the first step of stepped care, can be delivered effectively in routine clinical care. It led to a significant reduction of fatigue severity. Patients who received I-CBT in the MHCs did not profit less from the intervention than those in the tertiary treatment center. However, in the RCT, there were no missing values, whereas in the MHCs, post–I-CBT data of one-fifth of the patients had to be imputed. As treatment outcome for research dropouts may not be the most favorable, it is possible that we overestimated the efficacy of implemented I-CBT. Our sensitivity analysis, in which we assumed that these patients are maximally fatigued, showed a smaller reduction of fatigue in the MHCs, falling below the benchmark.

An important finding is that one-fifth of the patients did not start with I-CBT but directly commenced with face-to-face CBT. In the benchmark study, almost all patients started with I-CBT. Nevertheless, we can assume that patients who did not want I-CBT did not participate in an RCT testing it, which is a general limitation of testing I-CBT in an RCT. It is known that 15.5% of the patients eligible to enter the RCT refused because they preferred face-to-face CBT. An important issue for the interpretation of our results is that we cannot know how many patients who started directly with face-to-face CBT would have improved in I-CBT. They were probably not a random sample of all eligible patients. If this subgroup, for example, already expected to profit less from I-CBT, not including them might have had led to inflated results of I-CBT. Likewise, if these were mainly patients of therapists with little confidence in I-CBT, who, therefore, less convincingly offered it, they might have been better off in face-to-face CBT, which also would have led to inflated results of I-CBT after implementation.

According to therapist reports, about 30% of patients who started I-CBT dropped out during I-CBT. Although this figure is informative, it should be noted that therapist reports may not be the most reliable indication of dropout in I-CBT [9]. Furthermore, of the patients who were still severely fatigued and impaired after implemented I-CBT, about 1 in 5 stepped up to face-to-face CBT. In comparison, in the tertiary treatment center, approximately half of the patients stepped up when still fatigued or impaired after I-CBT. In the RCT, it was found that stepping up generally led to additional therapy gains. This could partly explain why in the RCT, patients had a larger decrease in fatigue severity: relatively more patients in the RCT received both I-CBT and face-to-face CBT. The problem of not starting and not stepping up is common in stepped care [37,38].

Another study [39] compared the outcome of stepped care for CFS in routine clinical care (ie, MHC) with the outcome of CBT in a tertiary treatment center. The first step was CBT using a self-help booklet with therapist guidance via email, and the second step was face-to-face CBT. Compared with outcomes in the tertiary treatment center, the MHC showed a lower reduction of fatigue severity and, contrary to our study, also a lower increase in physical functioning. Interestingly, previous studies found that the guided self-help, as well as the face-to-face CBT, could be delivered as effectively in the MHC as in the context of an RCT [5,6,26]. However, in combination with face-to-face CBT, the results were less positive. This suggests that there is something specific to stepped care that makes it more difficult to deliver in routine clinical care than face-to-face CBT, I-CBT, or guided self-help alone [39]. Several explanations could be considered. Delivering face-to-face CBT after a minimal intervention with limited results may demand more treatment experience from therapists than starting face-to-face CBT from the start, also because the patients in need of face-to-face CBT may be relatively complex to treat [6]. In our study, therapists were more experienced, and training had paid special attention to these difficulties specific to stepped care. Still, it seems that despite the training, many patients did not step up after unsuccessful I-CBT.

In the comparison of routine clinical care and care in a tertiary treatment center, it is important to consider possible differences between patients of both settings that may influence outcome. For CFS, it was found in routine clinical care that samples more often comprised patients with psychiatric comorbidity [26], which might negatively influence outcome [40]. Furthermore, we anticipated the inclusion of patients with ICF, because MHCs treat these as well, as they too benefit from CBT [22]. Interestingly, our study showed when only selecting patients who met CDC criteria for CFS, the reduction of fatigue in the MHCs did fall within the benchmark (whereas when selecting the total group, this fell below the benchmark). Our post hoc analysis showed that patients with ICF did not profit less than patients with CFS, but the group of patients for whom the diagnosis (ICF or CFS) was unknown had significantly lower treatment outcome.

We evaluated the influence of therapist factors on treatment outcome. In accordance with a former study investigating the role of the therapist on treatment outcome after implementation [17], we found considerable variance in outcome between therapists. We expected that therapists’ attitudes toward eHealth and manualized treatment would influence treatment outcome. Unfortunately, because of the limited number of patients per therapist, we were unable to perform the multilevel analysis we planned, which is a limitation of the study. The correlations between therapists’ attitudes and fatigue reduction were in the expected direction, but none was significant. Replication with more patients per therapist is needed, as it is likely that we lacked statistical power because of the small sample size.

### Strengths and Limitations

A limitation of the study is that the moments on which feedback was given during the I-CBT were not exactly the same as in the benchmark study. In the latter study, patients had received either I-CBT with feedback on prescheduled moments (*protocol-driven therapist feedback*) or I-CBT with feedback only when the patient asked for it (*on demand*) [8,9]. As both treatment arms did not differ in outcome, both were combined to calculate the benchmark. In this study, therapists were advised to give feedback according to the feedback schedule used in the *protocol-driven* condition of the benchmark study, but during supervision, it became clear that therapists did not always follow the schedule.

Furthermore, step-up criteria differed slightly. Additional face-to-face CBT was offered in both studies when patients were still severely fatigued and impaired following I-CBT. In the benchmark study, the Sickness Impact Profile 8 was used to assess the level of disability, but it was too lengthy for use in the MHCs. Therefore, the SF-36 was used to assess whether severe limitations in physical or social functioning were present. Although both instruments are used to assess limitations in functioning, it is possible that a different subgroup of patients were selected to step up using the 2 measures.

Another limitation is that we do not have information on usage of the program, either for patients or for therapists. For example, it is not recorded when therapist feedback was provided. Furthermore, the duration of the face-to-face CBT and the number of sessions was not registered.

Finally, the data collection was, to warrant patient privacy, done by the therapists, and questionnaires were sometimes scored by hand. This procedure may have reduced the reliability of data collection.

There were some important strengths as well. First, our study not only evaluated I-CBT in routine clinical care but also shed light on how I-CBT can be embedded in routine care. Second, by comparing the results with a benchmark, we were able to put the results in perspective. Finally, we included multiple treatment centers, across the Netherlands, which contributed to ecological validity of our study.

### Future Directions

The success of I-CBT and stepped care in routine clinical care can probably be improved by aiming at avoiding dropout and increasing the numbers of patients stepping up. To avoid dropout, it would be important to know why patients dropped out. Information on usage of the program would be helpful. If, for example dropout occurs more during 1 treatment module than another, that specific module could be improved. It is also important to know how aspects of therapist guidance (quantity and perceived quality) influenced dropout to improve the therapist training.

Likewise, to encourage stepping up, it is important to know what prevented patients from stepping up. The main reasons given were that patients did not want CBT anymore or were unable to start at that moment. More knowledge about reasons for not wanting CBT is needed to develop strategies to increase the number of patients who step up. If, for example, the long duration of treatment is a reason, one could evaluate the effect of I-CBT sooner, for example, after 3 or 4 months, to step up earlier. This would shorten the duration of the total treatment and may help avoid the loss of motivation to step up [9].

Furthermore, other options to embed I-CBT in routine care should be considered to increase the number of patients who profit from treatment—by offering matched care, for example. Unfortunately, it is not known which patients profit from I-CBT and which patients would profit more from face-to-face CBT. Future research should search for predictors of the outcome of different treatment formats or determine if following patient preference leads to better results. Therapists and patients may be well capable of resolving together what treatment form would work best for the patient. I-CBT and face-to-face CBT are both based on the same treatment principles and protocol, and it is mainly the form of communication that differs. It would be interesting to compare CBT offered as stepped care with preferred care, that is, either I-CBT or face-to-face CBT, depending on the preference of the patient. It was found in a meta-analysis that treatment outcome is higher when the patient receives the treatment of preference [41].

Finally, it is important to also investigate long-term outcome of implemented stepped care. This may be difficult to achieve in an observational multicenter study but would be a valuable contribution. A recent study showed that positive outcome for CFS after CBT is only partially maintained at long-term follow-up up to 10 years after treatment, as 37% still had fatigue within normal ranges [3]. Although efforts should be made to increase this proportion, it confirms the notion that one can recover from CFS and maintain the gains. We cannot assume that the long-term outcome for face-to-face CBT is the same as I-CBT/stepped care. It may, for example, be possible that the interventions at the end of the therapy (goal reaching, evaluation, and preparing the future) are important to retain the accomplishments, although probably a larger proportion of patients did not reach these modules.

### Conclusions

This is, to our knowledge, the first study to evaluate I-CBT for CFS, embedded in stepped-care, in routine clinical care. I-CBT was as effective as in tertiary care and could be embedded in routine clinical care where additional face-to-face CBT was offered if needed. Increasing the number of patients who step up after I-CBT is the most important remaining issue for implementation of stepped care.

## Appendix

Multimedia Appendix 1 Decision letter from the medical ethical committee.

## Abbreviations

CBT: cognitive behavioral therapy
CDC: Centers for Disease Control and Prevention
CFS: chronic fatigue syndrome
CIS: Checklist Individual Strength
eHealth: electronic health
I-CBT: internet-based cognitive behavioral therapy
ICF: idiopathic chronic fatigue
MHC: mental health care center
RCI: Reliable Change Index
RCT: randomized controlled trial
SF-36: Short Form-36
WSAS: Work and Social Adjustment Scale
