# Supplementary material for: Internet-Based Cognitive Behavioral Therapy for Chronic Fatigue Syndrome Integrated in Routine Clinical Care: Implementation Study
Source: J Med Internet Res. 2019 Oct 10;21(10):e14037. doi: 10.2196/14037 (PMC6914231; doi:10.2196/14037)
Supplement: Multimedia Appendix 1 [file jmir_v21i10e14037_app1.pdf]

Worm-Smeitink, Margreet

Van: Cruysen, Yvonne van der namens Postbus Commissie Mensgebonden Onderzoek
Verzonden: donderdag 6 juni 2019 11:38
Aan: 'Margreet Worm'
CC: Worm-Smeitink, Margreet
Onderwerp: 2014-1236 Niet WMO verklaring Engels

Title: Grip op vermoeidheid
Filenumber CMO : 2014-1236

Dear mrs. Worm - Smeitink,

On behalf of the research ethics committee of the Radboud University Nijmegen Medical Centre I hereby let you know that the abovementioned study doesn't fall within the remit of the Medical Research Involving Human Subjects Act (WMO).

The study has been reviewed by the ethics committee on the basis of the Dutch Code of conduct for health research, the Dutch Code of conduct for responsible use, the Dutch Personal Data Protection Act and the Medical Treatment Agreement Act.

The ethics committee has passed a positive judgment on the study.

The documents mentioned in appendix 1 have been reviewed.

Best regards,
Prof. dr. P.N.R Dekhuijzen, Chairman

Research Ethics Committee
Radboud University Nijmegen Medical Centre

Concernstaf Kwaliteit en Veiligheid
Commissie Mensgebonden Onderzoek
T (024) 361 31 54

Radboud universitair medisch centrum
Postbus 9101, 6500 HB Nijmegen (huispost 628)
Geert Grooteplein (route 628)
www.radboudumc.nl
www.cmoregio-a-n.nl

Appendix 1:

- Aanbiedingsbrief versie 1 d.d. 6 augustus 2014
- Protocol Grip op vermoeidheid versie 1
- Informatie voor cliënt versie 1 d.d. 16 september 2014
- Vragenlijst EuroQol-6d
- Vragenlijst PCQ
- Vragenlijst MCQ
- Reactie op commissievragen d.d. 16 september 2014
